# The Developmental Origins of Opioid Use Disorder and Its Comorbidities

**DOI:** 10.3389/fnhum.2021.601905

**Published:** 2021-02-11

**Authors:** Sophia C. Levis, Stephen V. Mahler, Tallie Z. Baram

**Affiliations:** ^1^Department of Anatomy and Neurobiology, University of California, Irvine, Irvine, CA, United States; ^2^Department of Neurobiology and Behavior, University of California, Irvine, Irvine, CA, United States; ^3^Department of Pediatrics, University of California, Irvine, Irvine, CA, United States

**Keywords:** early life stress, opioids, addiction, sex, anhedonia, reward, circuit, extinction

## Abstract

Opioid use disorder (OUD) rarely presents as a unitary psychiatric condition, and the comorbid symptoms likely depend upon the diverse risk factors and mechanisms by which OUD can arise. These factors are heterogeneous and include genetic predisposition, exposure to prescription opioids, and environmental risks. Crucially, one key environmental risk factor for OUD is early life adversity (ELA). OUD and other substance use disorders are widely considered to derive in part from abnormal reward circuit function, which is likely also implicated in comorbid mental illnesses such as depression, bipolar disorder, and schizophrenia. ELA may disrupt reward circuit development and function in a manner predisposing to these disorders. Here, we describe new findings addressing the effects of ELA on reward circuitry that lead to OUD and comorbid disorders, potentially *via* shared neural mechanisms. We discuss some of these OUD-related problems in both humans and animals. We also highlight the increasingly apparent, crucial contribution of biological sex in mediating the range of ELA-induced disruptions of reward circuitry which may confer risk for the development of OUD and comorbid neuropsychiatric disorders.

## Introduction

Opioid use disorder (OUD) is a growing epidemic in the United States and globally. To mitigate the rise in opioid-related morbidity and mortality, effective strategies are urgently needed to prevent the onset of opioid addiction by identifying individuals at high risk for developing OUD. Notably, OUD often occurs with psychiatric comorbidities such as depression, bipolar disorder, and schizophrenia (Brooner et al., 1997), all of which involve dysfunctional reward processing. Therefore, studying the basis for this disruption will provide greater understanding and insight into treating both OUD and its comorbidities.

The risk factors for OUD are numerous and complex, and genetics (Kreek et al., 2012; Crist et al., 2019; Jiang et al., 2019), drug availability (Volkow et al., 2011; Wright et al., 2014), and environmental factors such as early life adversity (ELA; Dube et al., 2003; Sinha, 2008; Kreek et al., 2012) all play a role. ELA related to poverty, trauma and chaotic environment affects over 30% of children in the U.S. (American Psychological Association, 2018). ELA is linked to numerous long-term negative health consequences including obesity, heart disease, respiratory illnesses, as well as cognitive and emotional problems (Felitti et al., 1998), and it is associated with several affective problems that indicate dysfunction of the brain’s reward circuitry (Kessler et al., 1997, 2010; Anda et al., 2006; Green et al., 2010; Pechtel and Pizzagalli, 2011; Novick et al., 2018). While a variety of the physical and mental health outcomes following ELA may lead to enhanced risk for OUD and its many comorbidities, here we focus on the effects of ELA on reward-related behaviors and underlying circuitry and propose that disrupted reward processing is a common developmental mechanism by which OUD and its comorbidities may arise following ELA. We also highlight the contribution of biological sex to the range of outcomes related to ELA-induced aberrations in reward circuitry.

## Normal Reward Circuit Development Involves An Early-Life Sensitive Period

Reward circuitry in the brain is a network comprised of cortical and subcortical forebrain structures that regulate reward seeking. This circuitry is evolutionarily adapted to drive the acquisition of natural rewards, such as food, water, and reproduction. However, the maladaptive function of this circuitry can also lead to psychiatric manifestations such as mood disorders and addiction.

Whereas the reward circuitry has been extensively studied in the adolescent and mature brain, its function and developmental trajectory in infancy and early childhood are less well-known. The ventral tegmental area (VTA), nucleus accumbens (NAc), and amygdala, major nodes of the reward circuit, begin to appear in the first trimester in humans and around the second week of gestation in rodents, and continue to undergo significant maturation postnatally (Birnie et al., 2020). Behavioral manifestations of the reward function, such as responsivity to sucrose (Desor et al., 1973; Vigorito and Sclafani, 1988) and appetitive learning (Johanson and Hall, 1979; Hayne et al., 1986), emerge within the first months of life in humans and within the first postnatal days in rodents. These developmental timelines suggest that reward circuitry in a rodent in its first week of life might approximate that of a human neonate (Birnie et al., 2020).

The development of these circuits that occurs early in postnatal life suggests a possible sensitive period during which time aberrant environmental signals, such as parental abuse or neglect, may shape their developmental trajectories (Baram et al., 2012; Glynn and Baram, 2019; Luby et al., 2020). Analogous influences of critical environmental signals on network maturation are known for other circuits, including the visual and auditory (Zhang et al., 2001; Li et al., 2006). Just as these systems require predictable sensory inputs at specific times during development to mature properly, parental signals may provide important stimuli for the maturing reward system (Hane and Fox, 2016; Davis et al., 2017; Andersen, 2018; Glynn and Baram, 2019). Thus, understanding how the early environment alters reward circuitry will be critical for developing future interventions against OUD and other mental health problems.

## Dysfunction of Reward Circuits: A Common Thread for Oud and Its Comorbidities?

The high prevalence of multiple diagnoses in patients with OUD (Kessler, 2004) supports shared or overlapping underlying processes and has led to searches for common genetic mechanisms (Carey et al., 2016). OUD is often diagnosed in patients who have other mental health problems (Brooner et al., 1997; Conway et al., 2006; Farrugia et al., 2011; Danovitch, 2016). Dysfunction of reward circuitry has been implicated in many of these other mental health diagnoses, such as depression and bipolar disorder (Russo and Nestler, 2013; Pizzagalli, 2014; Whitton et al., 2015), post-traumatic stress disorder (PTSD; Nawijn et al., 2015), personality disorders (Lawrence et al., 2010; Murray et al., 2018), and schizophrenia or psychosis (Kapur et al., 2005; Radua et al., 2015; Whitton et al., 2015). The specific comorbidities present with OUD also appear to be mediated by gender (Brooner et al., 1997; Conway et al., 2006). While women with OUD are more likely to also have a diagnosis of mood, anxiety, and eating disorders, men are more likely to have a diagnosed personality disorder (Brooner et al., 1997).

Notably, the prevalence of dual diagnoses is particularly high among patients who have experienced ELA, suggesting that ELA may impact a shared substrate involved in OUD and its comorbidities. In a study of patients admitted for chemical dependency treatment, those who reported a history of childhood abuse were also more likely to show symptoms of other reward-related comorbidities such as depression, bipolar, and anxiety disorders (Ellason et al., 1996). Another study found a very high co-incidence of PTSD and opioid abuse among women that was explained by a history of childhood trauma (Najavits et al., 1997). The risk for schizophrenia and psychosis is also increased by ELA (van Os et al., 2010; Bentall et al., 2014), which are highly comorbid with substance use disorder (Schmidt L. M. et al., 2011; Li et al., 2020). Palatable food cravings and disordered eating are strongly associated with ELA (Halmi, 2009; Dallman, 2014; Osadchiy et al., 2019), and these cravings are commonly observed in individuals with OUD (Morabia et al., 1989; Pelchat, 2002; Mysels and Sullivan, 2010; Canan et al., 2017; McDonald and Laurent, 2019; Nolan, 2019). This high co-incidence of multiple reward-related problems suggests a common underlying mechanism by which disruption of reward circuitry may lead to a variety of poor mental health outcomes.

## Developmental Origins: Ela Leads to Poor Neuropsychiatric Health Outcomes

Numerous studies have linked ELA to poor cognitive (Lupien et al., 2009; Pechtel and Pizzagalli, 2011; Chen and Baram, 2016; Short and Baram, 2019) and emotional health (Heim and Nemeroff, 2001; Anda et al., 2006; Smyke et al., 2007; Maccari et al., 2014; Callaghan and Tottenham, 2016; Hane and Fox, 2016; Krugers et al., 2016; Strathearn et al., 2020). For example, ELA is associated with lower educational achievement (Shonkoff et al., 2012) and poorer executive functioning abilities (McDermott et al., 2012). Evidence from clinical and epidemiological literature demonstrate links between adverse childhood experiences and increased risk for depression, anxiety, PTSD, eating disorders, and psychosis (Felitti et al., 1998; Chapman et al., 2004; Whitfield et al., 2005; Anda et al., 2006; Bale et al., 2010). The specific psychiatric outcomes resulting from ELA also vary by gender (Humphreys et al., 2015), with women more frequently diagnosed with anxiety and depression (Hammen et al., 2000; Heim and Nemeroff, 2001; Davis and Pfaff, 2014), whereas men are more likely to be diagnosed with personality disorders after ELA (Anda et al., 2006), the same pattern seen among those with comorbid OUD (Brooner et al., 1997).

Adverse childhood experiences are also robustly associated with later-life substance addiction (Nurco et al., 1996; Simpson and Miller, 2002; Dube et al., 2003; Widom et al., 2006; Gershon et al., 2008; Sinha, 2008; Enoch, 2011; Shand et al., 2011; Stein et al., 2017; Marsh et al., 2018). Results from the Adverse Childhood Experiences study show that ELA can increase the risk for injection drug use up to 11-fold (Anda et al., 2006) and that ELA increases the likelihood of early initiation of drug use independent of availability or changes in social attitudes towards drugs (Dube et al., 2003), suggesting a specific effect of adverse experiences on addiction liability. Additionally, individuals with a history of ELA are more likely to be prescribed opioid pain medications (Anda et al., 2008). This effect was mediated by an increased likelihood to experience other health and psychosocial problems, which highlights the interplay among the numerous physical and mental health problems associated with ELA, and the challenges in discerning causal mechanisms.

Interestingly, women appear to be particularly predisposed to OUD following ELA (Gershon et al., 2008; Lansford et al., 2010; Shand et al., 2011; Marsh et al., 2018). For example, although men have higher rates of overall substance dependence diagnoses, women who have experienced ELA are overrepresented among heroin and nonmedical prescription opioid users (Shand et al., 2011; Marsh et al., 2018). Women diagnosed with OUD are also two to three times more likely to have a history of PTSD related to ELA than men with OUD (Najavits et al., 1997). While this could be accounted for by the fact that girls tend to experience more childhood trauma than boys (Felitti et al., 1998), the magnitude of difference suggests a mediating role of sex. The type of adversity experienced may also interact with biological sex to affect outcomes. For example, Shand et al. (2011) found that emotional neglect during childhood predicted drug dependence in women, whereas PTSD predicted drug-related diagnoses for men. Again, the presence of other comorbidities varied by sex; men were more likely to display antisocial behaviors, whereas women were more likely to be diagnosed with anxiety and depression. These differences suggest divergent mechanisms by which ELA may alter reward circuit development between sexes, resulting in psychiatric outcomes that differ between men and women.

## Anhedonia and Oud, Each Manifestations of Reward Circuit Dysfunction, Arise After Ela

The paragraphs above suggest a strong association between ELA and malfunction of the reward circuit, which can manifest as OUD or other problems in reward-related behaviors. Many of these are common across several mental illnesses and may share common biological substrates. Anhedonia defined broadly as an inability to experience pleasure is a feature of substance use disorder in some individuals (Ahmed and Koob, 1998; Koob and Moal, 2001; Janiri et al., 2005; Hatzigiakoumis et al., 2011; Sussman and Leventhal, 2014; Kiluk et al., 2019; Brenner et al., 2020) and of other psychiatric diagnoses that are comorbid with addiction (Gorwood, 2008), such as depression (Loas, 1996; Blanchard et al., 2001; Pizzagalli et al., 2008; Martinotti et al., 2012), schizophrenia and psychosis (Andreasen and Olsen, 1982; Blanchard et al., 2001; Martinotti et al., 2012), PTSD (Risbrough et al., 2018), eating disorders (Davis and Woodside, 2002; Halmi, 2009), and other “high-risk” behaviors (Franken et al., 2006).

Indeed, the concept of anhedonia serves as a distinct useful transdiagnostic construct for understanding the role of altered reward processing in the etiology of psychiatric conditions (Bedwell et al., 2014; Lake et al., 2017). In line with the Research Domain Criteria (RDoC) framework put forth by the NIH, the ability to define a neurobiological basis of anhedonia, along with empirical behavioral measures both in humans and animal models, makes anhedonia a useful translational construct for studying reward circuit dysfunction and related behavioral disorders such as those seen after ELA (Cuthbert and Insel, 2013). Furthermore, the ubiquity of anhedonia as a feature of many of the psychiatric outcomes of ELA provides evidence that a mechanism by which ELA may impact cognitive and emotional health outcomes is through disruption of reward circuit development (Birnie et al., 2020). There are multiple domains of anhedonic behaviors that can be measured in humans and animal models which may have distinct neural processes (Der-Avakian and Markou, 2012; Shankman et al., 2014; Zald and Treadway, 2017). For example, anhedonia may represent a deficit in either anticipatory or consummatory reward, motivation, can be manifest for some reinforcers but not others (e.g., social vs. food rewards), and is also described as a feature of flat affect (for review, see Shankman et al., 2014). The neural substrates that govern these different forms of anhedonia have been explored (Gorwood, 2008; Der-Avakian and Markou, 2012; Treadway and Zald, 2013; Pizzagalli, 2014), and the specific effects of ELA on distinct types of anhedonic behaviors as well as their potentially dissociable neural substrates is an important area of continued investigation.

## How Does Ela Provoke Anhedonia, Oud, and Comorbidities? A Need for Animal Studies

While studies in humans offer important insights into the effects of ELA on reward circuitry, one cannot dissociate the influence of early-life experiences on reward circuitry function from other genetic and environmental variables that may mediate the links between ELA, OUD, and other comorbidities. Animal models provide a method for investigating the effects of these environmental factors in isolation.

In animal studies, several different models of ELA have been used to isolate the effects of adversity on brain development from other genetic and environmental variables. These methods, such as maternal separation (MS), limited bedding and nesting (LBN), fostering by abusive caregivers, and others, have been extensively described elsewhere (Molet et al., 2014; Doherty et al., 2017; Walker et al., 2017; Wakeford et al., 2018; Brenhouse and Bath, 2019). In rodents and non-human primates, numerous studies have demonstrated that ELA results in behavioral phenotypes that suggest underlying dysfunction in reward-related brain regions (Molet et al., 2014; Andersen, 2015, 2018; Wakeford et al., 2018; Bonapersona et al., 2019; Birnie et al., 2020). The particular behavioral outcomes of ELA in animal models can vary depending on the type, timing, and duration of the paradigm, the species and strain of animal, and the timing and type of behavioral assays (Schmidt M. V. et al., 2011; Molet et al., 2014; Andersen, 2015; Walker et al., 2017; Brenhouse and Bath, 2019; Demaestri et al., 2020; Lundberg et al., 2020), as well as sex (Kundakovic et al., 2013; Bath, 2020). While this poses a challenge for interpreting this vast literature, the variability also mirrors human experience; indeed, ELA in humans can take many different forms, such as poverty, trauma, physical or sexual abuse, and neglect, and these, in combination with other environmental and biological factors, likely contribute to individual differences in clinical outcomes (Shand et al., 2011; Daskalakis et al., 2013; Sheridan and McLaughlin, 2014; Strathearn et al., 2020), highlighting the sensitivity of the brain to different types of stressors during these developmental periods.

Given that anhedonia has been associated clinically with many of the psychiatric outcomes of ELA, establishing whether ELA can actually *cause* anhedonia seems useful for determining neurobiological mechanisms that may ultimately underlie ELA-associated OUD and its comorbidities. Thus, we will highlight some animal studies that have focused specifically on anhedonia. The expression of anhedonia in animal models appears to be mediated by interactions between the ELA paradigm, biological sex, and testing parameters (Matthews and Robbins, 2003; Rüedi-Bettschen et al., 2005; Der-Avakian and Markou, 2010; Leussis et al., 2012; Lukkes et al., 2017; Di Segni et al., 2019). For example, in male rodents, ELA imposed *via* rearing for 1 week (P2-P9) in cages with limited bedding and nesting materials (LBN) leads to enduring anhedonia for both natural and drug rewards. This includes blunted sucrose and palatable food preference, reduced interest in social play, and decreased low-effort cocaine consumption (Molet et al., 2016; Bolton et al., 2018a, b). In contrast, such anhedonia is not observed in female rats after LBN (Levis et al., 2019). Yet, others have identified an age-dependent reduction of sucrose preference and depressive-like behaviors in female mice (Goodwill et al., 2019). Using a MS model of ELA, both male and female rats have reduced sucrose preference later in life (Matthews et al., 1996; Leventopoulos et al., 2009; Coccurello et al., 2014). Anhedonia has been reported also in nonhuman primates exposed to maternal deprivation and maltreatment (Rosenblum and Paully, 1987; Paul et al., 2000; Pryce et al., 2004; Kaufman et al., 2007; Glynn and Baram, 2019), such as reduced sucrose preference (Paul et al., 2000) or interest in social interaction (Coplan et al., 1996). However, others have found increased sucrose drinking in juvenile males (Nelson et al., 2009).

In contrast to natural reward anhedonia, other studies have demonstrated increased sensitivity to drug-related rewards (Andersen, 2018) as well as addiction-related behavioral traits (Hynes et al., 2018) after ELA. Although this may appear contradictory, these findings support the notion that the behavioral expression of altered reward circuitry by ELA depends on reward type and testing paradigm; thus, anhedonia and reward-seeking are not necessarily mutually exclusive.

**Figure 1:**
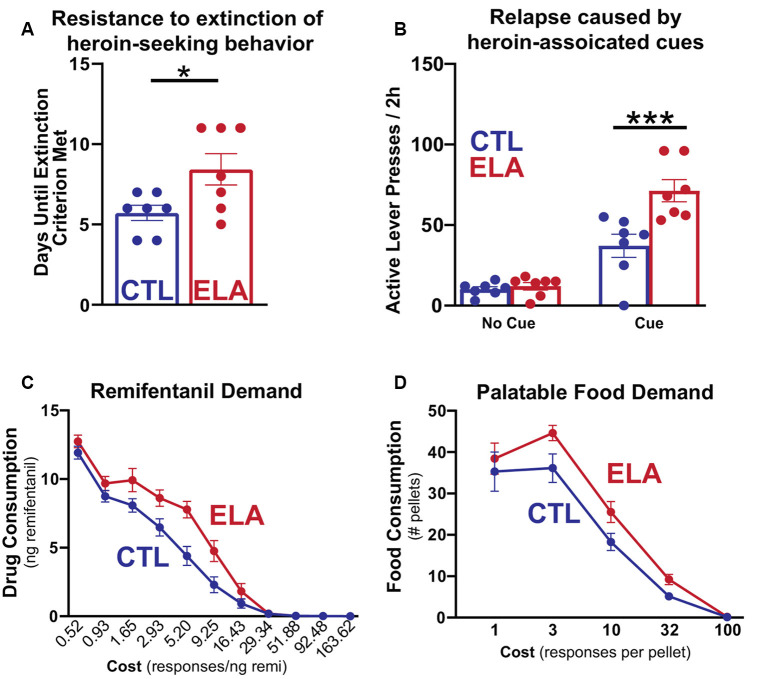
Early life adversity (ELA) augments opioid-seeking behaviors and increases the demand for opioid drugs and highly palatable food. Adapted from Figures 1, 2 in Levis et al. (2019). **(A)** Female LBN-experienced (ELA) rats trained to self-administer intravenous heroin engage in more persistent heroin-seeking behavior after the withdrawal of the drug and **(B)** augmented relapse induced by heroin-associated cues than their control (CTL) counterparts. **(C,D)** On an economic task measuring sensitivity to increasing cost to obtain the desired reward, ELA rats are willing to exert more effort to access both opioid drug and food rewards at a higher cost than controls. This indicates increased demand for opioids and highly palatable food that is relatively insensitive to high costs. *p < 0.05; ***p < 0.001.

While effects of ELA on increased alcohol and cocaine-seeking have been extensively studied and reviewed (Andersen, 2018), considerably less work has been done to model the effects of ELA specifically on opioid addiction vulnerability. Some evidence exists that MS increases morphine seeking in both male and female adult rats (Abad et al., 2016; Mohammadian et al., 2019) while others observed morphine preference only in MS males (Kalinichev et al., 2002; Vazquez et al., 2005, 2006; Michaels and Holtzman, 2008; Vey et al., 2016). Holtzman and colleagues show that male rats that have experienced MS demonstrate a greater place preference for morphine than their control counterparts (Michaels and Holtzman, 2008) and increased locomotor sensitization to repeated morphine, a measure of the psychoactive properties of the drug (Kalinichev et al., 2002). However, others have found attenuated sensitivity to the rewarding properties of heroin in MS females (Matthews and Robbins, 2003). Using the LBN model of ELA, we have demonstrated that, while males develop anhedonia for natural rewards like social play, palatable food, and sucrose (Molet et al., 2016; Bolton et al., 2018a, b), females developed a strikingly different phenotype (Figure 1). LBN females exhibit a marked increase in addiction-like seeking for opioid drugs (Levis et al., 2019). These rats were resistant to the extinction of opioid-seeking behavior, had stronger cue-induced and heroin-primed reinstatement responses, and increased motivation to self-administer the opioid remifentanil in a translationally relevant task measuring economic demand (Treadway et al., 2009; Bentzley et al., 2013; Bickel et al., 2014), demonstrating a motivation to obtain the drug even at a very high cost. Motivation for consuming palatable food was also significantly higher in LBN females, concurrent with the marked increase in addiction-like seeking for opioid drugs. Notably, this same phenomenon has been observed among patients seeking treatment for OUD (McDonald and Laurent, 2019).

Together, the findings in rodents and non-human primates suggest that ELA disrupts the maturation of reward circuits, and the resulting behavioral manifestations may vary by the timing, duration, and nature of the ELA and be further modulated by sex. Whereas deficits in reward-seeking behaviors are observed in males, such deficits are not commonly found in females. Rather, in females, the prevailing phenotype includes the enhanced consumption of opioids (and other drugs of abuse) and palatable food. The mechanisms underlying this phenotype are poorly understood and may involve ELA-induced changes in both reward and stress circuits. Support for this notion is provided by studies showing that female rats that have experienced stress tend to engage in more pro-hedonic consumption of palatable food (Dallman et al., 2003, 2005; Pecoraro et al., 2004; Jahng, 2011, 2014; Tomiyama et al., 2011; Machado et al., 2013; Kim et al., 2015), and that this may be specifically associated with anhedonia (Jahng et al., 2012; Jahng, 2014). Much information is needed to gain insight into the bases of palatable food craving as sex-dependent comorbidity of OUD.

Furthermore, the variable consequences of ELA on distinct assays of reward-seeking behaviors in animal models demonstrate that reward processing is not a singular phenomenon; rather, individuals may express different and dissociable phenotypes that suggest potentially discrete mechanisms of reward circuit disruption. Thus, further investigation into how ELA alters specific aspects of reward processing and underlying neural substrates will be critical for understanding the biological processes that contribute to the risk for OUD and comorbid disorders.

## How Might Ela Lead to Oud and Related Disorders? Evidence from Clinical Imaging Studies

Evidence from human imaging studies suggests impaired development of specific reward-related brain regions and circuits after ELA that impose a risk for substance abuse and related comorbidities. Many studies have demonstrated functional and neuroanatomical effects of ELA on brain regions involved with reward and reward-learning, such as the hippocampus, amygdala, medial prefrontal cortex, and striatal areas including nucleus accumbens (Bremner, 2003; Hackman and Farah, 2009; Rao et al., 2010; Pechtel and Pizzagalli, 2011; Gee et al., 2013; Boecker et al., 2014; Callaghan and Tottenham, 2016; Teicher et al., 2016; Miguel et al., 2019; Herzberg and Gunnar, 2020). Childhood maltreatment is associated with blunted activation of these brain regions during reward processing tasks (Dillon et al., 2009; Mehta et al., 2010; Goff et al., 2013; Novick et al., 2018), a potential functional mechanism explaining the presence of anhedonia among individuals who have experienced ELA. Of these, the striatum appears to be especially important in mediating the link between reduced reward reactivity and ELA (Dillon et al., 2009; Goff et al., 2013; Goff and Tottenham, 2015; Egerton et al., 2016; Kamkar et al., 2017; Dennison et al., 2019). The ventral striatum in particular seems to be a key mediator between ELA, anhedonia, and substance abuse. Corral-Frías et al. (2015) report that reduced reward reactivity in the ventral striatum predicts ELA-associated anhedonia and structural equation modeling revealed that this relationship also predicts substance-related coping behaviors, such as self-medication. This finding highlights a possible common mechanism by which ELA can lead to OUD and its comorbidities. The type of adversity experienced may also mediate the striatal response to reward (Dennison et al., 2019; Herzberg and Gunnar, 2020), as ELA in the form of childhood poverty, specifically, is associated with increased reactivity to reward in the striatum (Gonzalez et al., 2016), especially in girls (Romens et al., 2015). These sex- and experience-dependent differences are consistent with the observed variability of mental health outcomes in humans and behavioral phenotypes in animals.

## Ela Causes Functional and Anatomical Changes in Reward-Related Brain Regions: Evidence from Animal Models

Building on clinical evidence, studies using animal models provide tools for identifying mechanisms that underlie disruptions in reward circuitry after ELA. In analogy to human literature, these outcomes appear to be partially mediated by sex. In males, our group has previously shown that anhedonia after LBN is associated with altered functional connectivity between the amygdala and mPFC in rats that may be mediated by CRH expression in the amygdala (Bolton et al., 2018a). This is supported by evidence that depressive-like behaviors and natural reward anhedonia following LBN are associated with disrupted amygdala-PFC and PFC-striatal functional connectivity (Yan et al., 2017). Additionally, Walker et al. (2017) have observed morphological and functional changes in the basolateral amygdala (BLA) and reduced functional connectivity between BLA and PFC in LBN-exposed male rats (Guadagno et al., 2018a, b). MS-induced ELA alters the development of PFC→NAc projections and dopamine (DA) signaling within the pathway in male rats (Brenhouse et al., 2013). In females, MS induces early maturation of the BLA-PFC circuit (Honeycutt et al., 2020), and early life social stress alters resting-state functional connectivity in NAc, hippocampus, and PFC (Nephew et al., 2017). In nonhuman primates, maltreatment during infancy leads to increased amygdala volume (Howell et al., 2014) and altered connectivity in regions implicated in mood disorders (Howell et al., 2013). c-Fos mapping studies measuring neuronal activity further suggest specific ELA-induced alterations in reward circuit function (Rincón-Cortés and Sullivan, 2016; Bolton et al., 2018a, b; Di Segni et al., 2019). Specifically, ELA leads to reduced NAc c-Fos activation in response to typically-rewarding stimuli like a social interaction (Rincón-Cortés and Sullivan, 2016), or aberrant over-activation of other regions associated with stress and reward (Bolton et al., 2018a, b).

Molecular mechanisms mediating the effects of ELA on OUD and related comorbidities may involve alterations in neurotransmitter and neuromodulator systems. Whereas a comprehensive discussion of this important topic is beyond the scope of this review article, a few salient points are mentioned: A vast literature documents the role of DA signaling in motivated and reward-seeking behaviors. Altered DA signaling is an important mediator of drug-seeking (Koob, 1992) as well as other psychiatric problems associated with ELA such as mood disorders (Diehl and Gershon, 1992) and psychosis (Kapur et al., 2005) and has been implicated in the expression of anhedonia (Willner et al., 1992; Pizzagalli, 2014). ELA has been extensively linked to dysfunction of the DA system in rodents, especially in the striatum (for a comprehensive review of this literature, see Bonapersona et al., 2018), and this may be mediated by alterations in other stress and reward-related transmitter systems (Forster et al., 2018). Additionally, the effects of early life experiences on DA signaling may be more pronounced in females (Camp et al., 1984; Chocyk et al., 2011). It is therefore tempting to speculate about the role of ELA-provoked deficits in DA signaling as involved in ELA-related OUD and its comorbidities.

Endogenous opioids play an important role in mediating hedonic processes (Smith and Berridge, 2007; Mahler and Berridge, 2009, 2012; Mitchell et al., 2018) as well as social attachment early in life (Panksepp et al., 1980), so the endogenous opioid system might also represent an important link between ELA and reward-related outcomes later in life. Alterations in opioid receptor mRNA have been observed in both males and females after ELA, although differentially between the sexes. Chang et al. (2019) show female-specific increases in NAc mu and delta-opioid receptor mRNA levels in mice after early life predator odor exposure. Nylander and colleagues have found long-term alterations in endogenous opioid peptides and opioid and DA receptor expression in reward-associated areas that vary both by sex and by the duration of MS (Ploj et al., 1999, 2001, 2003a,b; Ploj and Nylander, 2003; Gustafsson et al., 2008). Opioid receptors are known to modulate striatal DA signaling (Mulder et al., 1984; Johnson and North, 1992), an effect that may be potentiated by ELA (Karkhanis et al., 2016). Thus, disturbances in endogenous opioids might also mediate ELA-induced alterations of striatal DA signaling leading to aberrant reward-related behaviors. These ELA-induced opioids and DA-related disruptions suggest a mechanism by which ELA may lead simultaneously or in parallel to psychiatric disorders and enhanced consumption of opioids (Khantzian, 1987; Dallman et al., 2005; Kim et al., 2015; Lovallo et al., 2018).

Together with evidence from human subjects, these findings demonstrate that ELA alters important reward-related circuit nodes to provoke vulnerability to poor psychiatric outcomes. Establishing causality between network- and molecular-level changes induced by ELA and resulting reward-related deficits remains an important area of investigation to cure OUD and its psychiatric comorbidities.

## Conclusion

Evidence across species suggests that ELA during sensitive developmental periods alters the developmental trajectory of reward circuitry. The precise nature of ELA, the potentially disparate consequences of different types of ELA, and the mechanisms underlying the aberrant maturation of reward circuits remain topics of much-needed investigation. The resulting maladaptive reward processing is likely a mechanism common to OUD and its comorbidities. As both animal and human studies demonstrate, the manifestations of this aberrant reward circuit function are varied and depend on the type and extent of adversity, biological sex, and later life experiences. However, functional, anatomical, and molecular disruptions in reward-related brain regions such as the medial PFC, striatum, and amygdala have been described across multiple paradigms and several species, suggesting a common developmental origin. Likewise, anhedonia may be an important behavioral biomarker of disturbed reward processing that links ELA, OUD, and other mental health problems. Further investigation into the neurobiological basis for ELA-induced reward circuit disruptions will provide key insights into the origins of OUD and its comorbidities and may uncover new interventions that will be successful in treating both.

## Author Contributions

SCL drafted the review. SVM and TZB guided the writing process and provided revisions. All authors contributed to the article and approved the submitted version.

## Conflict of Interest

The authors declare that the research was conducted in the absence of any commercial or financial relationships that could be construed as a potential conflict of interest.

## References

[B1] Abad A. T.-K. Miladi-Gorji H. Bigdeli I. (2016). Effects of swimming exercise on morphine-induced reward and behavioral sensitization in maternally-separated rat pups in the conditioned place preference procedure. Neurosci. Lett. 631, 79–84. 10.1016/j.neulet.2016.08.011 27519931

[B2] Ahmed S. H. Koob G. F. (1998). Transition from moderate to excessive drug intake: change in hedonic set point. Science 282, 298–300. 10.1126/science.282.5387.298 9765157

[B3] American Psychological Association (2018). Stress in America: Generation Z. Stress in America^TM^ Survey. Washington, DC: American Psychiatric Association.

[B4] Anda R. F. Brown D. W. Felitti V. J. Dube S. R. Giles W. H. (2008). Adverse childhood experiences and prescription drug use in a cohort study of adult HMO patients. BMC Public Health 8:198. 10.1186/1471-2458-8-198 18533034 PMC2440750

[B5] Anda R. F. Felitti V. J. Bremner J. D. Walker J. D. Whitfield C. Perry B. D.. (2006). The enduring effects of abuse and related adverse experiences in childhood. A convergence of evidence from neurobiology and epidemiology. Eur. Arch. Psychiatry Clin. Neurosci. 256, 174–186. 10.1007/s00406-005-0624-4 16311898 PMC3232061

[B6] Andersen S. L. (2015). Exposure to early adversity: points of cross-species translation that can lead to improved understanding of depression. Dev. Psychopathol. 27, 477–491. 10.1017/S0954579415000103 25997766 PMC5237807

[B7] Andersen S. L. (2018). Stress, sensitive periods, and substance abuse. Neurobiol. Stress 10:100140. 10.1016/j.ynstr.2018.100140 30569003 PMC6288983

[B8] Andreasen N. C. Olsen S. (1982). Negative v positive schizophrenia: definition and validation. Arch. Gen. Psychiatry 39, 789–794. 10.1001/archpsyc.1982.04290070025006 7165478

[B9] Bale T. L. Baram T. Z. Brown A. S. Goldstein J. M. Insel T. R. McCarthy M. M.. (2010). Early life programming and neurodevelopmental disorders. Biol. Psychiatry 68, 314–319. 10.1016/j.biopsych.2010.05.028 20674602 PMC3168778

[B10] Baram T. Z. Davis E. P. Obenaus A. Sandman C. A. Small S. L. Solodkin A.. (2012). Fragmentation and unpredictability of early-life experience in mental disorders. Am. J. Psychiatry 169, 907–915. 10.1176/appi.ajp.2012.11091347 22885631 PMC3483144

[B11] Bath K. G. (2020). Synthesizing views to understand sex differences in response to early life adversity. Trends Neurosci. 43, 300–310. 10.1016/j.tins.2020.02.004 32353334 PMC7195459

[B12] Bedwell J. S. Gooding D. C. Chan C. C. Trachik B. J. (2014). Anhedonia in the age of RDoC. Schizophr. Res. 160, 226–227. 10.1016/j.schres.2014.10.028 25464922

[B13] Bentall R. P. de Sousa P. Varese F. Wickham S. Sitko K. Haarmans M.. (2014). From adversity to psychosis: pathways and mechanisms from specific adversities to specific symptoms. Soc. Psychiatry Psychiatr. Epidemiol. 49, 1011–1022. 10.1007/s00127-014-0914-0 24919446

[B14] Bentzley B. S. Fender K. M. Aston-Jones G. (2013). The behavioral economics of drug self-administration: a review and new analytical approach for within-session procedures. Psychopharmacology 226, 113–125. 10.1007/s00213-012-2899-2 23086021 PMC3572328

[B15] Bickel W. K. Johnson M. W. Koffarnus M. N. MacKillop J. Murphy J. G. (2014). The behavioral economics of substance use disorders: reinforcement pathologies and their repair. Annu. Rev. Clin. Psychol. 10, 641–677. 10.1146/annurev-clinpsy-032813-153724 24679180 PMC4501268

[B16] Birnie M. T. Kooiker C. L. Short A. K. Bolton J. L. Chen Y. Baram T. Z. (2020). Plasticity of the reward circuitry after early-life adversity: mechanisms and significance. Biol. Psychiatry 87, 875–884. 10.1016/j.biopsych.2019.12.018 32081365 PMC7211119

[B17] Blanchard J. L. Horan W. P. Brown S. A. (2001). Diagnostic differences in social anhedonia: a longitudinal study of schizophrenia and major depressive disorder. J. Abnorm. Psychol. 110, 363–371. 10.1037/0021-843x.110.3.363 11502079

[B18] Boecker R. Holz N. E. Buchmann A. F. Blomeyer D. Plichta M. M. Wolf I.. (2014). Impact of early life adversity on reward processing in young adults: EEG-fMRI results from a prospective study over 25 years. PLoS One 9:e104185. 10.1371/journal.pone.0104185 25118701 PMC4131910

[B19] Bolton J. L. Molet J. Regev L. Chen Y. Rismanchi N. Haddad E.. (2018a). Anhedonia following early-life adversity involves aberrant interaction of reward and anxiety circuits and is reversed by partial silencing of amygdala corticotropin-releasing hormone gene. Biol. Psychiatry 83, 137–147. 10.1016/j.biopsych.2017.08.023 29033027 PMC5723546

[B20] Bolton J. L. Ruiz C. M. Rismanchi N. Sanchez G. A. Castillo E. Huang J.. (2018b). Early-life adversity facilitates acquisition of cocaine self-administration and induces persistent anhedonia. Neurobiol. Stress 8, 57–67. 10.1016/j.ynstr.2018.01.002 29888304 PMC5991313

[B21] Bonapersona V. Joëls M. Sarabdjitsingh R. A. (2018). Effects of early life stress on biochemical indicators of the dopaminergic system: a 3 level meta-analysis of rodent studies. Neurosci. Biobehav. Rev. 95, 1–16. 10.1016/j.neubiorev.2018.09.003 30201218

[B22] Bonapersona V. Kentrop J. Van Lissa C. J. van der Veen R. Joëls M. Sarabdjitsingh R. A. (2019). The behavioral phenotype of early life adversity: a 3-level meta-analysis of rodent studies. Neurosci. Biobehav. Rev. 102, 299–307. 10.1016/j.neubiorev.2019.04.021 31047892

[B23] Bremner J. D. (2003). Functional neuroanatomical correlates of traumatic stress revisited 7 years later, this time with data. Psychopharmacol. Bull. 37, 6–25. 14566211

[B24] Brenhouse H. C. Bath K. G. (2019). Bundling the haystack to find the needle: challenges and opportunities in modeling risk and resilience following early life stress. Front. Neuroendocrinol. 54:100768. 10.1016/j.yfrne.2019.100768 31175880 PMC6708473

[B25] Brenhouse H. C. Lukkes J. L. Andersen S. L. (2013). Early life adversity alters the developmental profiles of addiction-related prefrontal cortex circuitry. Brain Sci. 3, 143–158. 10.3390/brainsci3010143 24961311 PMC4061828

[B26] Brenner P. Brandt L. Li G. DiBernardo A. Bodén R. Reutfors J. (2020). Substance use disorders and risk for treatment resistant depression: a population-based, nested case-control study. Addiction 115, 768–777. 10.1111/add.14866 31656053 PMC7078870

[B27] Brooner R. K. King V. L. Kidorf M. Schmidt C. W. Jr. Bigelow G. E. (1997). Psychiatric and substance use comorbidity among treatment-seeking opioid abusers. Arch. Gen. Psychiatry 54, 71–80. 10.1001/archpsyc.1997.01830130077015 9006403

[B28] Callaghan B. L. Tottenham N. (2016). The neuro-environmental loop of plasticity: a cross-species analysis of parental effects on emotion circuitry development following typical and adverse caregiving. Neuropsychopharmacology 41, 163–176. 10.1038/npp.2015.204 26194419 PMC4677125

[B29] Camp D. M. Robinson T. E. Becker J. B. (1984). Sex differences in the effects of early experience on the development of behavioral and brain asymmetries in rats. Physiol. Behav. 33, 433–439. 10.1016/0031-9384(84)90166-5 6514832

[B30] Canan F. Karaca S. Sogucak S. Gecici O. Kuloglu M. (2017). Eating disorders and food addiction in men with heroin use disorder: a controlled study. Eat. Weight Disord. 22, 249–257. 10.1007/s40519-017-0378-9 28434177

[B31] Carey C. E. Agrawal A. Bucholz K. K. Hartz S. M. Lynskey M. T. Nelson E. C.. (2016). Associations between polygenic risk for psychiatric disorders and substance involvement. Front. Genet. 7:149. 10.3389/fgene.2016.00149 27574527 PMC4983546

[B32] Chang L. Kigar S. L. Ho J. H. Cuarenta A. Gunderson H. C. Baldo B. A.. (2019). Early life stress alters opioid receptor mRNA levels within the nucleus accumbens in a sex-dependent manner. Brain Res. 1710, 102–108. 10.1016/j.brainres.2018.12.040 30594547 PMC6701935

[B33] Chapman D. P. Whitfield C. L. Felitti V. J. Dube S. R. Edwards V. J. Anda R. F. (2004). Adverse childhood experiences and the risk of depressive disorders in adulthood. J. Affect. Disord. 82, 217–225. 10.1016/j.jad.2003.12.013 15488250

[B34] Chen Y. Baram T. Z. (2016). Toward understanding how early-life stress reprograms cognitive and emotional brain networks. Neuropsychopharmacology 41, 197–206. 10.1038/npp.2015.181 26105143 PMC4677123

[B35] Chocyk A. Przyborowska A. Dudys D. Majcher I. Maćkowiak M. Wędzony K. (2011). The impact of maternal separation on the number of tyrosine hydroxylase-expressing midbrain neurons during different stages of ontogenesis. Neuroscience 182, 43–61. 10.1016/j.neuroscience.2011.03.008 21396433

[B36] Coccurello R. Bielawski A. Zelek-Molik A. Vetulani J. Kowalska M. D’Amato F. R.. (2014). Brief maternal separation affects brain α1-adrenoceptors and apoptotic signaling in adult mice. Prog. Neuropsychopharmacol. Biol. Psychiatry 48, 161–169. 10.1016/j.pnpbp.2013.10.004 24128685

[B37] Conway K. P. Compton W. Stinson F. S. Grant B. F. (2006). Lifetime comorbidity of DSM-IV mood and anxiety disorders and specific drug use disorders: results from the national epidemiologic survey on alcohol and related conditions. J. Clin. Psychiatry 67, 247–257. 10.4088/jcp.v67n0211 16566620

[B38] Coplan J. D. Andrews M. W. Rosenblum L. A. Owens M. J. Friedman S. Gorman J. M.. (1996). Persistent elevations of cerebrospinal fluid concentrations of corticotropin-releasing factor in adult nonhuman primates exposed to early-life stressors: implications for the pathophysiology of mood and anxiety disorders. Proc. Natl. Acad. Sci. U S A 93, 1619–1623. 10.1073/pnas.93.4.1619 8643680 PMC39991

[B39] Corral-Frías N. S. Nikolova Y. S. Michalski L. J. Baranger D. A. Hariri A. R. Bogdan R. (2015). Stress-related anhedonia is associated with ventral striatum reactivity to reward and transdiagnostic psychiatric symptomatology. Psychol. Med. 45, 2605–2617. 10.1017/S0033291715000525 25853627 PMC4700837

[B40] Crist R. C. Reiner B. C. Berrettini W. H. (2019). A review of opioid addiction genetics. Curr. Opin. Psychol. 27, 31–35. 10.1016/j.copsyc.2018.07.014 30118972 PMC6368898

[B41] Cuthbert B. N. Insel T. R. (2013). Toward the future of psychiatric diagnosis: the seven pillars of RDoC. BMC Med. 11:126. 10.1186/1741-7015-11-126 23672542 PMC3653747

[B42] Dallman M. F. (2014). Early life stress: nature and nurture. Endocrinology 155, 1569–1572. 10.1210/en.2014-1267 24746330

[B43] Dallman M. F. Pecoraro N. Akana S. F. la Fleur S. E. Gomez F. Houshyar H.. (2003). Chronic stress and obesity: a new view of “comfort food”. Proc. Natl. Acad. Sci. U S A 100, 11696–11701. 10.1073/pnas.1934666100 12975524 PMC208820

[B44] Dallman M. F. Pecoraro N. C. la Fleur S. E. (2005). Chronic stress and comfort foods: self-medication and abdominal obesity. Brain Behav. Immun. 19, 275–280. 10.1016/j.bbi.2004.11.004 15944067

[B45] Danovitch I. (2016). Post-traumatic stress disorder and opioid use disorder: a narrative review of conceptual models. J. Addict. Dis. 35, 169–179. 10.1080/10550887.2016.1168212 27010975

[B46] Daskalakis N. P. Bagot R. C. Parker K. J. Vinkers C. H. de Kloet E. R. (2013). The three-hit concept of vulnerability and resilience: toward understanding adaptation to early-life adversity outcome. Psychoneuroendocrinology 38, 1858–1873. 10.1016/j.psyneuen.2013.06.008 23838101 PMC3773020

[B48] Davis E. P. Pfaff D. (2014). Sexually dimorphic responses to early adversity: implications for affective problems and autism spectrum disorder. Psychoneuroendocrinology 49, 11–25. 10.1016/j.psyneuen.2014.06.014 25038479 PMC4165713

[B49] Davis E. P. Stout S. A. Molet J. Vegetabile B. Glynn L. M. Sandman C. A.. (2017). Exposure to unpredictable maternal sensory signals influences cognitive development across species. Proc. Natl. Acad. Sci. U S A 114, 10390–10395. 10.1073/pnas.1703444114 28893979 PMC5625898

[B47] Davis C. Woodside D. B. (2002). Sensitivity to the rewarding effects of food and exercise in the eating disorders. Compr. Psychiatry 43, 189–194. 10.1053/comp.2002.32356 11994836

[B50] Demaestri C. Pan T. Critz M. Ofray D. Gallo M. Bath K. G. (2020). Type of early life adversity confers differential, sex-dependent effects on early maturational milestones in mice. Horm. Behav. 124:104763. 10.1016/j.yhbeh.2020.104763 32407728 PMC7487052

[B51] Dennison M. J. Rosen M. L. Sambrook K. A. Jenness J. L. Sheridan M. A. McLaughlin K. A. (2019). Differential associations of distinct forms of childhood adversity with neurobehavioral measures of reward processing: a developmental pathway to depression. Child Dev. 90, e96–e113. 10.1111/cdev.13011 29266223 PMC6013316

[B52] Der-Avakian A. Markou A. (2010). Neonatal maternal separation exacerbates the reward-enhancing effect of acute amphetamine administration and the anhedonic effect of repeated social defeat in adult rats. Neuroscience 170, 1189–1198. 10.1016/j.neuroscience.2010.08.002 20691770 PMC2949486

[B53] Der-Avakian A. Markou A. (2012). The neurobiology of anhedonia and other reward-related deficits. Trends Neurosci. 35, 68–77. 10.1016/j.tins.2011.11.005 22177980 PMC3253139

[B54] Desor J. A. Maller O. Turner R. E. (1973). Taste in acceptance of sugars by human infants. J. Comp. Physiol. Psychol. 84, 496–501. 10.1037/h0034906 4745817

[B55] Di Segni M. Andolina D. D’Addario S. L. Babicola L. Ielpo D. Luchetti A.. (2019). Sex-dependent effects of early unstable post-natal environment on response to positive and negative stimuli in adult mice. Neuroscience 413, 1–10. 10.1016/j.neuroscience.2019.06.016 31228589

[B56] Diehl D. J. Gershon S. (1992). The role of dopamine in mood disorders. Compr. Psychiatry 33, 115–120. 10.1016/0010-440x(92)90007-d 1347497

[B57] Dillon D. G. Holmes A. J. Birk J. L. Brooks N. Lyons-Ruth K. Pizzagalli D. A. (2009). Childhood adversity is associated with left basal ganglia dysfunction during reward anticipation in adulthood. Biol. Psychiatry 66, 206–213. 10.1016/j.biopsych.2009.02.019 19358974 PMC2883459

[B58] Doherty T. S. Blaze J. Keller S. M. Roth T. L. (2017). Phenotypic outcomes in adolescence and adulthood in the scarcity-adversity model of low nesting resources outside the home cage. Dev. Psychobiol. 59, 703–714. 10.1002/dev.21547 28767135 PMC5569321

[B59] Dube S. R. Felitti V. J. Dong M. Chapman D. P. Giles W. H. Anda R. F. (2003). Childhood abuse, neglect, and household dysfunction and the risk of illicit drug use: the adverse childhood experiences study. Pediatrics 111, 564–572. 10.1542/peds.111.3.564 12612237

[B60] Egerton A. Valmaggia L. R. Howes O. D. Day F. Chaddock C. A. Allen P.. (2016). Adversity in childhood linked to elevated striatal dopamine function in adulthood. Schizophr. Res. 176, 171–176. 10.1016/j.schres.2016.06.005 27344984 PMC5147458

[B61] Ellason J. W. Ross C. A. Sainton K. Mayran L. W. (1996). Axis I and II comorbidity and childhood trauma history in chemical dependency. Bull. Menninger Clin. 60, 39–51. 8742671

[B62] Enoch M.-A. (2011). The role of early life stress as a predictor for alcohol and drug dependence. Psychopharmacology 214, 17–31. 10.1007/s00213-010-1916-6 20596857 PMC3005022

[B63] Farrugia P. L. Mills K. L. Barrett E. Back S. E. Teesson M. Baker A.. (2011). Childhood trauma among individuals with co-morbid substance use and post-traumatic stress disorder. Mental Health Subst. Use 4, 314–326. 10.1080/17523281.2011.598462 21984884 PMC3188414

[B64] Felitti V. J. Anda R. F. Nordenberg D. F. Williamson D. F. Spitz A. M. Edwards V.. (1998). Relationship of childhood abuse and household dysfunction to many of the leading causes of death in adults: the adverse childhood experiences (ACE) study. Am. J. Prev. Med. 14, 245–258. 10.1016/s0749-3797(98)00017-8 9635069

[B65] Forster G. L. Anderson E. M. Scholl J. L. Lukkes J. L. Watt M. J. (2018). Negative consequences of early-life adversity on substance use as mediated by corticotropin-releasing factor modulation of serotonin activity. Neurobiol. Stress 9, 29–39. 10.1016/j.ynstr.2018.08.001 30151419 PMC6108067

[B66] Franken I. H. Zijlstra C. Muris P. (2006). Are nonpharmacological induced rewards related to anhedonia? A study among skydivers. Prog. Neuropsychopharmacol. Biol. Psychiatry 30, 297–300. 10.1016/j.pnpbp.2005.10.011 16303225

[B67] Gee D. G. Gabard-Durnam L. J. Flannery J. Goff B. Humphreys K. L. Telzer E. H.. (2013). Early developmental emergence of human amygdala-prefrontal connectivity after maternal deprivation. Proc. Natl. Acad. Sci. U S A 110, 15638–15643. 10.1073/pnas.1307893110 24019460 PMC3785723

[B68] Gershon A. Minor K. Hayward C. (2008). Gender, victimization, and psychiatric outcomes. Psychol. Med. 38, 1377–1391. 10.1017/S0033291708003000 18387212 PMC3102516

[B69] Glynn L. M. Baram T. Z. (2019). The influence of unpredictable, fragmented parental signals on the developing brain. Front. Neuroendocrinol. 53:100736. 10.1016/j.yfrne.2019.01.002 30711600 PMC6776465

[B71] Goff B. Gee D. G. Telzer E. H. Humphreys K. L. Gabard-Durnam L. Flannery J.. (2013). Reduced nucleus accumbens reactivity and adolescent depression following early-life stress. Neuroscience 249, 129–138. 10.1016/j.neuroscience.2012.12.010 23262241 PMC3646076

[B70] Goff B. Tottenham N. (2015). Early-life adversity and adolescent depression: mechanisms involving the ventral striatum. CNS Spectr. 20, 337–345. 10.1017/S1092852914000674 25511634 PMC5928787

[B72] Gonzalez M. Z. Allen J. P. Coan J. A. (2016). Lower neighborhood quality in adolescence predicts higher mesolimbic sensitivity to reward anticipation in adulthood. Dev. Cogn. Neurosci. 22, 48–57. 10.1016/j.dcn.2016.10.003 27838595 PMC5275766

[B73] Goodwill H. L. Manzano-Nieves G. Gallo M. Lee H.-I. Oyerinde E. Serre T.. (2019). Early life stress leads to sex differences in development of depressive-like outcomes in a mouse model. Neuropsychopharmacology 44, 711–720. 10.1038/s41386-018-0195-5 30188513 PMC6372611

[B74] Gorwood P. (2008). Neurobiological mechanisms of anhedonia. Dialogues Clin. Neurosci. 10, 291–299. 10.31887/DCNS.2008.10.3/pgorwood 18979942 PMC3181880

[B75] Green J. McLaughlin K. A. Berglund P. A. Gruber M. J. Sampson N. A. Zaslavsky A. M.. (2010). Childhood adversities and adult psychiatric disorders in the national comorbidity survey replication I: associations with first onset of DSM-IV disorders. Arch. Gen. Psychiatry 67, 113–123. 10.1001/archgenpsychiatry.2009.186 20124111 PMC2822662

[B76] Guadagno A. Kang M. S. Devenyi G. A. Mathieu A. P. Rosa-Neto P. Chakravarty M.. (2018a). Reduced resting-state functional connectivity of the basolateral amygdala to the medial prefrontal cortex in preweaning rats exposed to chronic early-life stress. Brain Struct. Funct. 223, 3711–3729. 10.1007/s00429-018-1720-3 30032360

[B77] Guadagno A. Wong T. P. Walker C.-D. (2018b). Morphological and functional changes in the preweaning basolateral amygdala induced by early chronic stress associate with anxiety and fear behavior in adult male, but not female rats. Prog. Neuropsychopharmacol. Biol. Psychiatry 81, 25–37. 10.1016/j.pnpbp.2017.09.025 28963066

[B78] Gustafsson L. Oreland S. Hoffmann P. Nylander I. (2008). The impact of postnatal environment on opioid peptides in young and adult male Wistar rats. Neuropeptides 42, 177–191. 10.1016/j.npep.2007.10.006 18082882

[B79] Hackman D. A. Farah M. J. (2009). Socioeconomic status and the developing brain. Trends Cogn. Sci. 13, 65–73. 10.1016/j.tics.2008.11.003 19135405 PMC3575682

[B80] Halmi K. A. (2009). Perplexities and provocations of eating disorders. J. Child Psychol. Psychiatry 50, 163–169. 10.1111/j.1469-7610.2008.01983.x 19220599

[B81] Hammen C. Henry R. Daley S. (2000). Depression and sensitization to stressors among young women as a function of childhood adversity. J. Consult. Clin. Psychol. 68, 782–787. 10.1037/0022-006x.68.5.782 11068964

[B82] Hane A. A. Fox N. A. (2016). Early caregiving and human biobehavioral development: a comparative physiology approach. Curr. Opin. Behav. Sci. 7, 82–90. 10.1016/j.cobeha.2015.12.002 26753173 PMC4703360

[B83] Hatzigiakoumis D. S. Martinotti G. Di Giannantonio M. Janiri L. (2011). Anhedonia and substance dependence: clinical correlates and treatment options. Front. Psychiatry 2:10. 10.3389/fpsyt.2011.00010 21556280 PMC3089992

[B84] Hayne H. Greco C. Earley L. Griesler P. Rovee-Collier C. (1986). Ontogeny of early event memory: II. Encoding and retrieval by 2- and 3-month-olds. Inf. Behav. Dev. 9, 461–472. 10.1016/0163-6383(86)90018-4

[B85] Heim C. Nemeroff C. B. (2001). The role of childhood trauma in the neurobiology of mood and anxiety disorders: preclinical and clinical studies. Biol. Psychiatry 49, 1023–1039. 10.1016/s0006-3223(01)01157-x 11430844

[B86] Herzberg M. P. Gunnar M. R. (2020). Early life stress and brain function: activity and connectivity associated with processing emotion and reward. NeuroImage 209:116493. 10.1016/j.neuroimage.2019.116493 31884055 PMC7056544

[B87] Honeycutt J. A. Demaestri C. Peterzell S. Silveri M. M. Cai X. Kulkarni P.. (2020). Altered corticolimbic connectivity reveals sex-specific adolescent outcomes in a rat model of early life adversity. eLife 9:e52651. 10.7554/eLife.52651 31958061 PMC7010412

[B88] Howell B. R. Grand A. P. McCormack K. M. Shi Y. LaPrarie J. L. Maestripieri D.. (2014). Early adverse experience increases emotional reactivity in juvenile rhesus macaques: relation to amygdala volume. Dev. Psychobiol. 56, 1735–1746. 10.1002/dev.21237 25196846 PMC4433484

[B89] Howell B. R. McCormack K. M. Grand A. P. Sawyer N. T. Zhang X. Maestripieri D.. (2013). Brain white matter microstructure alterations in adolescent rhesus monkeys exposed to early life stress: associations with high cortisol during infancy. Biol. Mood Anxiety Disord. 3:21. 10.1186/2045-5380-3-21 24289263 PMC3880213

[B90] Humphreys K. L. Gleason M. M. Drury S. S. Miron D. Nelson C. A. III. Fox N. A.. (2015). Effects of institutional rearing and foster care on psychopathology at age 12 years in Romania: follow-up of an open, randomised controlled trial. Lancet Psychiatry 2, 625–634. 10.1016/S2215-0366(15)00095-4 26303560 PMC4550037

[B91] Hynes T. J. Thomas C. S. Zumbusch A. S. Samson A. Petriman I. Mrdja U.. (2018). Early life adversity potentiates expression of addiction-related traits. Prog. Neuropsychopharmacol. Biol. Psychiatry 87, 56–67. 10.1016/j.pnpbp.2017.09.005 28899646

[B92] Jahng J. W. (2011). An animal model of eating disorders associated with stressful experience in early life. Horm. Behav. 59, 213–220. 10.1016/j.yhbeh.2010.11.010 21093444

[B93] Jahng J. W. (2014). “Neural basis of anhedonia associated with stress-induced eating disorders,” in Anhedonia: A Comprehensive Handbook Volume I, ed. Ritsner M. (Dordrecht: Springer), 309–329.

[B94] Jahng J. W. Yoo S. B. Ryu V. Lee J. H. (2012). Hyperphagia and depression-like behavior by adolescence social isolation in female rats. Int. J. Dev. Neurosci. 30, 47–53. 10.1016/j.ijdevneu.2011.10.001 22027618

[B95] Janiri L. Martinotti G. Dario T. Reina D. Paparello F. Pozzi G.. (2005). Anhedonia and substance-related symptoms in detoxified substance-dependent subjects: a correlation study. Neuropsychobiology 52, 37–44. 10.1159/000086176 15942262

[B96] Jiang S. Kamei N. Bolton J. L. Ma X. Stern H. S. Baram T. Z.. (2019). Intra-individual methylomics detects the impact of early-life adversity. Life Sci. Alliance 2:e201800204. 10.26508/lsa.201800204 30936186 PMC6445397

[B97] Johanson I. Hall W. (1979). Appetitive learning in 1-day-old rat pups. Science 205, 419–421. 10.1126/science.451612 451612

[B98] Johnson S. North R. (1992). Opioids excite dopamine neurons by hyperpolarization of local interneurons. J. Neurosci. 12, 483–488. 10.1523/JNEUROSCI.12-02-00483.1992 1346804 PMC6575608

[B99] Kalinichev M. Easterling K. W. Holtzman S. G. (2002). Early neonatal experience of Long-Evans rats results in long-lasting changes in reactivity to a novel environment and morphine-induced sensitization and tolerance. Neuropsychopharmacology 27, 518–533. 10.1016/S0893-133X(02)00326-3 12377389

[B100] Kamkar N. H. Lewis D. J. van den Bos W. Morton J. B. (2017). Ventral striatal activity links adversity and reward processing in children. Dev. Cogn. Neurosci. 26, 20–27. 10.1016/j.dcn.2017.04.002 28436832 PMC6987763

[B101] Kapur S. Mizrahi R. Li M. (2005). From dopamine to salience to psychosis—linking biology, pharmacology and phenomenology of psychosis. Schizophr. Res. 79, 59–68. 10.1016/j.schres.2005.01.003 16005191

[B102] Karkhanis A. N. Rose J. H. Weiner J. L. Jones S. R. (2016). Early-life social isolation stress increases kappa opioid receptor responsiveness and downregulates the dopamine system. Neuropsychopharmacology 41, 2263–2274. 10.1038/npp.2016.21 26860203 PMC4946054

[B103] Kaufman D. Banerji M. A. Shorman I. Smith E. L. Coplan J. D. Rosenblum L. A.. (2007). Early-life stress and the development of obesity and insulin resistance in juvenile bonnet macaques. Diabetes 56, 1382–1386. 10.2337/db06-1409 17470564

[B104] Kessler R. C. (2004). The epidemiology of dual diagnosis. Biol. Psychiatry 56, 730–737. 10.1016/j.biopsych.2004.06.034 15556117

[B105] Kessler R. C. Davis C. G. Kendler K. S. (1997). Childhood adversity and adult psychiatric disorder in the US national comorbidity survey. Psychol. Med. 27, 1101–1119. 10.1017/s0033291797005588 9300515

[B106] Kessler R. C. McLaughlin K. A. Green J. G. Gruber M. J. Sampson N. A. Zaslavsky A. M.. (2010). Childhood adversities and adult psychopathology in the WHO World Mental Health Surveys. Br. J. Psychiatry 197, 378–385. 10.1192/bjp.bp.110.080499 21037215 PMC2966503

[B107] Khantzian E. J. (1987). “The self-medication hypothesis of addictive disorders: focus on heroin and cocaine dependence,” in The Cocaine Crisis, ed. Allen D. F. (Boston, MA: Springer), 65–74. 10.1176/ajp.142.11.1259 3904487

[B108] Kiluk B. D. Yip S. W. DeVito E. E. Carroll K. M. Sofuoglu M. (2019). Anhedonia as a key clinical feature in the maintenance and treatment of opioid use disorder. Clin. Psychol. Sci. 7, 1190–1206. 10.1177/2167702619855659 32042509 PMC7009780

[B109] Kim J. Y. Lee J.-H. Kim D. Kim S.-M. Koo J. Jahng J. W. (2015). Beneficial effects of highly palatable food on the behavioral and neural adversities induced by early life stress experience in female rats. Int. J. Biol. Sci. 11, 1150–1159. 10.7150/ijbs.12044 26327809 PMC4551751

[B110] Koob G. F. (1992). “Dopamine, addiction and reward,” in Seminars in Neuroscience ed Robbins T. W. (Cambridge, MA: Elsevier Inc), 139–148.

[B111] Koob G. F. Moal M. (2001). Drug addiction, dysregulation of reward, and allostasis. Neuropsychopharmacology 24, 97–129. 10.1016/S0893-133X(00)00195-0 11120394

[B112] Kreek M. Levran O. Reed B. Schlussman S. D. Zhou Y. Butelman E. R. (2012). Opiate addiction and cocaine addiction: underlying molecular neurobiology and genetics. J. Clin. Invest. 122, 3387–3393. 10.1172/JCI60390 23023708 PMC3534165

[B113] Krugers H. J. Arp J. M. Xiong H. Kanatsou S. Lesuis S. L. Korosi A.. (2016). Early life adversity: lasting consequences for emotional learning. Neurobiol. Stress 6, 14–21. 10.1016/j.ynstr.2016.11.005 28229105 PMC5314442

[B114] Kundakovic M. Lim S. Gudsnuk K. Champagne F. A. (2013). Sex-specific and strain-dependent effects of early life adversity on behavioral and epigenetic outcomes. Front. Psychiatry 4:78. 10.3389/fpsyt.2013.00078 23914177 PMC3730082

[B115] Lake J. I. Yee C. M. Miller G. A. (2017). Misunderstanding RDoC. Z. Psychol. 225, 170–174. 10.1027/2151-2604/a000301 31080700 PMC6510511

[B116] Lansford J. E. Dodge K. A. Pettit G. S. Bates J. E. (2010). Does physical abuse in early childhood predict substance use in adolescence and early adulthood? Child Maltreat. 15, 190–194. 10.1177/1077559509352359 20019026 PMC2868928

[B117] Lawrence K. A. Allen J. S. Chanen A. M. (2010). Impulsivity in borderline personality disorder: reward-based decision-making and its relationship to emotional distress. J. Pers. Disord. 24, 785–799. 10.1521/pedi.2010.24.6.785 21158600

[B118] Leussis M. P. Freund N. Brenhouse H. C. Thompson B. S. Andersen S. L. (2012). Depressive-like behavior in adolescents after maternal separation: sex differences, controllability, and GABA. Dev. Neurosci. 34, 210–217. 10.1159/000339162 22776911 PMC5267293

[B119] Leventopoulos M. Russig H. Feldon J. Pryce C. R. Opacka-Juffry J. (2009). Early deprivation leads to long-term reductions in motivation for reward and 5-HT1A binding and both effects are reversed by fluoxetine. Neuropharmacology 56, 692–701. 10.1016/j.neuropharm.2008.12.005 19138691

[B120] Levis S. C. Bentzley B. S. Molet J. Bolton J. L. Perrone C. R. Baram T. Z.. (2019). On the early life origins of vulnerability to opioid addiction. Mol. Psychiatry [Epub ahead of print]. 10.1038/s41380-019-0628-5 31822817 PMC7282971

[B121] Li K. J. Chen A. DeLisi L. E. (2020). Opioid use and schizophrenia. Curr. Opin. Psychiatry 33, 219–224. 10.1097/YCO.0000000000000593 32073422

[B122] Li Y. Fitzpatrick D. White L. E. (2006). The development of direction selectivity in ferret visual cortex requires early visual experience. Nat. Neurosci. 9, 676–681. 10.1038/nn1684 16604068

[B123] Loas G. (1996). Vulnerability to depression: a model centered on anhedonia. J. Affect. Disord. 41, 39–53. 10.1016/0165-0327(96)00065-1 8938204

[B124] Lovallo W. R. Acheson A. Vincent A. S. Sorocco K. H. Cohoon A. J. (2018). Early life adversity diminishes the cortisol response to opioid blockade in women: studies from the family health patterns project. PLoS One 13:e0205723. 10.1371/journal.pone.0205723 30312327 PMC6185842

[B125] Luby J. L. Baram T. Z. Rogers C. E. Barch D. M. (2020). Neurodevelopmental optimization after early-life adversity: cross-species studies to elucidate sensitive periods and brain mechanisms to inform early intervention. Trends Neurosci. 43, 744–751. 10.1016/j.tins.2020.08.001 32863044 PMC7530018

[B126] Lukkes J. L. Meda S. Thompson B. S. Freund N. Andersen S. L. (2017). Early life stress and later peer distress on depressive behavior in adolescent female rats: effects of a novel intervention on GABA and D2 receptors. Behav. Brain Res. 330, 37–45. 10.1016/j.bbr.2017.04.053 28499915 PMC5546234

[B127] Lundberg S. Nylander I. Roman E. (2020). Behavioral profiling in early adolescence and early adulthood of male wistar rats after short and prolonged maternal separation. Front. Behav. Neurosci. 14:37. 10.3389/fnbeh.2020.00037 32265671 PMC7096550

[B128] Lupien S. J. McEwen B. S. Gunnar M. R. Heim C. (2009). Effects of stress throughout the lifespan on the brain, behavior and cognition. Nat. Rev. Neurosci. 10, 434–445. 10.1038/nrn2639 19401723

[B129] Maccari S. Krugers H. J. Morley-Fletcher S. Szyf M. Brunton P. J. (2014). The consequences of early-life adversity: neurobiological, behavioral and epigenetic adaptations. J. Neuroendocrinol. 26, 707–723. 10.1111/jne.12175 25039443

[B130] Machado T. D. Dalle Molle R. Laureano D. P. Portella A. K. Werlang I. C. R. Benetti C. D. S.. (2013). Early life stress is associated with anxiety, increased stress responsivity and preference for “comfort foods” in adult female rats. Stress 16, 549–556. 10.3109/10253890.2013.816841 23781957

[B131] Mahler S. V. Berridge K. C. (2009). Which cue to “want?” Central amygdala opioid activation enhances and focuses incentive salience on a prepotent reward cue. J. Neurosci. 29, 6500–6513. 10.1523/JNEUROSCI.3875-08.2009 19458221 PMC2802210

[B132] Mahler S. V. Berridge K. C. (2012). What and when to “want”? Amygdala-based focusing of incentive salience upon sugar and sex. Psychopharmacology 221, 407–426. 10.1007/s00213-011-2588-6 22167254 PMC3444284

[B133] Marsh J. C. Park K. Lin Y. A. Bersamira C. (2018). Gender differences in trends for heroin use and nonmedical prescription opioid use, 2007–2014. J. Subst. Abuse Treat 87, 79–85. 10.1016/j.jsat.2018.01.001 29433788 PMC9084392

[B134] Martinotti G. Hatzigiakoumis D. Vita O. Clerici M. Petruccelli F. di Giannantonio M. (2012). Anhedonia and reward system: psychobiology, evaluation, and clinical features. Int. J. Clin. Med. 3, 697–713. 10.4236/ijcm.2012.37125

[B136] Matthews K. Hall F. S. Wilkinson L. S. Robbins T. W. (1996). Retarded acquisition and reduced expression of conditioned locomotor activity in adult rats following repeated early maternal separation: effects of prefeeding,d-amphetamine, dopamine antagonists and clonidine. Psychopharmacology 126, 75–84. 10.1007/BF02246414 8853220

[B135] Matthews K. Robbins T. W. (2003). Early experience as a determinant of adult behavioral responses to reward: the effects of repeated maternal separation in the rat. Neurosci. Biobehav. Rev. 27, 45–55. 10.1016/s0149-7634(03)00008-3 12732222

[B137] McDermott J. M. Westerlund A. Zeanah C. H. Nelson C. A. Fox N. A. (2012). Early adversity and neural correlates of executive function: implications for academic adjustment. Dev. Cogn. Neurosci. 2, S59–S66. 10.1016/j.dcn.2011.09.008 22682911 PMC3408020

[B138] McDonald E. Laurent J. (2019). Hedonic eating behaviors and food preferences associated with medication-assisted treatment for opioid use disorder. J. Opioid. Manag. 15, 487–494. 10.5055/jom.2019.0539 31850510

[B139] Mehta M. A. Gore-Langton E. Golembo N. Colvert E. Williams S. C. R. Sonuga-Barke E. (2010). Hyporesponsive reward anticipation in the basal ganglia following severe institutional deprivation early in life. J. Cogn. Neurosci. 22, 2316–2325. 10.1162/jocn.2009.21394 19929329

[B140] Michaels C. C. Holtzman S. G. (2008). Early postnatal stress alters place conditioning to both μ- and κ-opioid agonists. J. Pharmacol. Exp. Ther. 325, 313–318. 10.1124/jpet.107.129908 18203949

[B141] Miguel P. M. Pereira L. O. Silveira P. P. Meaney M. J. (2019). Early environmental influences on the development of children’s brain structure and function. Dev. Med. Child Neurol. 61, 1127–1133. 10.1111/dmcn.14182 30740660

[B142] Mitchell M. R. Berridge K. C. Mahler S. V. (2018). Endocannabinoid-enhanced “liking” in nucleus accumbens shell hedonic hotspot requires endogenous opioid signals. Cannabis Cannabinoid Res. 3, 166–170. 10.1089/can.2018.0021 30069500 PMC6069591

[B143] Mohammadian J. Najafi M. Miladi-Gorji H. (2019). Effect of enriched environment during adolescence on spatial learning and memory, and voluntary consumption of morphine in maternally separated rats in adulthood. Dev. Psychobiol. 61, 615–625. 10.1002/dev.21808 30488421

[B144] Molet J. Heins K. Zhuo X. Mei Y. T. Regev L. Baram T. Z.. (2016). Fragmentation and high entropy of neonatal experience predict adolescent emotional outcome. Transl. Psychiatry 6:e702. 10.1038/tp.2015.200 26731439 PMC5068874

[B145] Molet J. Maras P. M. Avishai-Eliner S. Baram T. Z. (2014). Naturalistic rodent models of chronic early-life stress. Dev. Psychobiol. 56, 1675–1688. 10.1002/dev.21230 24910169 PMC4777289

[B146] Morabia A. Fabre J. Ghee E. Zeger S. Orsat E. Robert A. (1989). Diet and Opiate addiction: a quantitative assessment of the diet of non-institutionalized opiate addicts. Br. J. Addict. 84, 173–180. 10.1111/j.1360-0443.1989.tb00566.x 2720181

[B147] Mulder A. H. Wardeh G. Hogenboom F. Frankhuyzen A. L. (1984). κ and δ-opioid receptor agonists differentially inhibit striatal dopamine and acetylcholine release. Nature 308, 278–280. 10.1038/308278a0 6322011

[B148] Murray L. Waller R. Hyde L. W. (2018). A systematic review examining the link between psychopathic personality traits, antisocial behavior, and neural reactivity during reward and loss processing. Personal. Disord. 9, 497–509. 10.1037/per0000308 30080060 PMC7238432

[B149] Mysels D. J. Sullivan M. A. (2010). The relationship between opioid and sugar intake: review of evidence and clinical applications. J. Opioid. Manag. 6, 445–452. 10.5055/jom.2010.0043 21269006 PMC3109725

[B150] Najavits L. M. Weiss R. D. Shaw S. R. (1997). The link between substance abuse and posttraumatic stress disorder in women: a research review. Am. J. Addict. 6, 273–283. 10.3109/10550499709005058 9398925

[B151] Nawijn L. van Zuiden M. Frijling J. L. Koch S. B. Veltman D. J. Olff M. (2015). Reward functioning in PTSD: a systematic review exploring the mechanisms underlying anhedonia. Neurosci. Biobehav. Rev. 51, 189–204. 10.1016/j.neubiorev.2015.01.019 25639225

[B152] Nelson E. E. Herman K. N. Barrett C. E. Noble P. L. Wojteczko K. Chisholm K.. (2009). Adverse rearing experiences enhance responding to both aversive and rewarding stimuli in juvenile rhesus monkeys. Biol. Psychiatry 66, 702–704. 10.1016/j.biopsych.2009.04.007 19450795 PMC3010188

[B153] Nephew B. C. Huang W. Poirier G. L. Payne L. King J. A. (2017). Altered neural connectivity in adult female rats exposed to early life social stress. Behav. Brain Res. 316, 225–233. 10.1016/j.bbr.2016.08.051 27594665 PMC6390839

[B154] Nolan L. J. (2019). Food selection, food craving, and body mass index in persons in treatment for substance use disorder. Appetite 138, 80–86. 10.1016/j.appet.2019.03.016 30880085

[B155] Novick A. M. Levandowski M. L. Laumann L. Philip N. S. Price L. H. Tyrka A. R. (2018). The effects of early life stress on reward processing. J. Psychiatr. Res. 101, 80–103. 10.1016/j.jpsychires.2018.02.002 29567510 PMC5889741

[B156] Nurco D. N. Kinlock T. W. O’Grady K. E. Hanlon T. E. (1996). Early family adversity as a precursor to narcotic addiction. Drug Alcohol Depend. 43, 103–113. 10.1016/s0376-8716(96)01299-9 8957149

[B157] Osadchiy V. Mayer E. A. Bhatt R. Labus J. S. Gao L. Kilpatrick L. A.. (2019). History of early life adversity is associated with increased food addiction and sex-specific alterations in reward network connectivity in obesity. Obes. Sci. Pract. 5, 416–436. 10.1002/osp4.362 31687167 PMC6819979

[B158] Panksepp J. Herman B. Vilberg T. Bishop P. DeEskinazi F. (1980). Endogenous opioids and social behavior. Neurosci. Biobehav. Rev. 4, 473–487. 10.1016/0149-7634(80)90036-6 6258111

[B159] Paul I. A. English J. A. Halaris A. (2000). Sucrose and quinine intake by maternally-deprived and control rhesus monkeys. Behav. Brain Res. 112, 127–134. 10.1016/s0166-4328(00)00173-x 10862943

[B160] Pechtel P. Pizzagalli D. A. (2011). Effects of early life stress on cognitive and affective function: an integrated review of human literature. Psychopharmacology 214, 55–70. 10.1007/s00213-010-2009-2 20865251 PMC3050094

[B161] Pecoraro N. Reyes F. Gomez F. Bhargava A. Dallman M. F. (2004). Chronic stress promotes palatable feeding, which reduces signs of stress: feedforward and feedback effects of chronic stress. Endocrinology 145, 3754–3762. 10.1210/en.2004-0305 15142987

[B162] Pelchat M. L. (2002). Of human bondage: food craving, obsession, compulsion, and addiction. Physiol. Behav. 76, 347–352. 10.1016/s0031-9384(02)00757-6 12117571

[B163] Pizzagalli D. A. (2014). Depression, stress and anhedonia: toward a synthesis and integrated model. Annu. Rev. Clin. Psychol. 10, 393–423. 10.1146/annurev-clinpsy-050212-185606 24471371 PMC3972338

[B164] Pizzagalli D. A. Iosifescu D. Hallett L. A. Ratner K. G. Fava M. (2008). Reduced hedonic capacity in major depressive disorder: evidence from a probabilistic reward task. J. Psychiatr. Res. 43, 76–87. 10.1016/j.jpsychires.2008.03.001 18433774 PMC2637997

[B165] Ploj K. Nylander I. (2003). Long-term effects on brain opioid and opioid receptor like-1 receptors after short periods of maternal separation in rats. Neurosci. Lett. 345, 195–197. 10.1016/s0304-3940(03)00515-9 12842289

[B166] Ploj K. Pham T. M. Bergström L. Mohammed A. H. Henriksson B. G. Nylander I. (1999). Neonatal handling in rats induces long-term effects on dynorphin peptides. Neuropeptides 33, 468–474. 10.1054/npep.1999.0764 10657526

[B169] Ploj K. Roman E. Bergström L. Nylander I. (2001). Effects of neonatal handling on nociceptin/orphanin FQ and opioid peptide levels in female rats. Pharmacol. Biochem. Behav. 69, 173–179. 10.1016/s0091-3057(01)00511-1 11420083

[B167] Ploj K. Roman E. Nylander I. (2003a). Long-term effects of maternal separation on ethanol intake and brain opioid and dopamine receptors in male wistar rats. Neuroscience 121, 787–799. 10.1016/s0306-4522(03)00499-8 14568037

[B168] Ploj K. Roman E. Nylander I. (2003b). Long-term effects of short and long periods of maternal separation on brain opioid peptide levels in male Wistar rats. Neuropeptides 37, 149–156. 10.1016/s0143-4179(03)00043-x 12860112

[B170] Pryce C. R. Dettling A. C. Spengler M. Schnell C. R. Feldon J. (2004). Deprivation of parenting disrupts development of homeostatic and reward systems in marmoset monkey offspring. Biol. Psychiatry 56, 72–79. 10.1016/j.biopsych.2004.05.002 15231438

[B171] Radua J. Schmidt A. Borgwardt S. Heinz A. Schlagenhauf F. McGuire P.. (2015). Ventral striatal activation during reward processing in psychosis: a neurofunctional meta-analysis. JAMA Psychiatry 72, 1243–1251. 10.1001/jamapsychiatry.2015.2196 26558708

[B172] Rao U. Chen L.-A. Bidesi A. S. Shad M. U. Thomas M. A. Hammen C. L. (2010). Hippocampal changes associated with early-life adversity and vulnerability to depression. Biol. Psychiatry 67, 357–364. 10.1016/j.biopsych.2009.10.017 20015483 PMC2821020

[B173] Rincón-Cortés M. Sullivan R. M. (2016). Emergence of social behavior deficit, blunted corticolimbic activity and adult depression-like behavior in a rodent model of maternal maltreatment. Translat. Psychiatry 6:e930. 10.1038/tp.2016.205 27779623 PMC5290349

[B174] Risbrough V. B. Glynn L. M. Davis E. P. Sandman C. A. Obenaus A. Stern H. S.. (2018). Does anhedonia presage increased risk of posttraumatic stress disorder? Adolescent anhedonia and posttraumatic disorders. Curr. Top. Behav. Neurosci. 38, 249–265. 10.1007/7854_2018_51 29796839 PMC9167566

[B175] Romens S. E. Casement M. D. McAloon R. Keenan K. Hipwell A. E. Guyer A. E.. (2015). Adolescent girls’ neural response to reward mediates the relation between childhood financial disadvantage and depression. J. Child Psychol. Psychiatry 56, 1177–1184. 10.1111/jcpp.12410 25846746 PMC4593710

[B176] Rosenblum L. A. Paully G. S. (1987). Primate models of separation-induced depression. Psychiatr. Clin. North Am. 10, 437–447. 10.1016/s0193-953x(18)30553-7 3120161

[B177] Rüedi-Bettschen D. Pedersen E. M. Feldon J. Pryce C. R. (2005). Early deprivation under specific conditions leads to reduced interest in reward in adulthood in Wistar rats. Behav. Brain Res. 156, 297–310. 10.1016/j.bbr.2004.06.001 15582116

[B178] Russo S. J. Nestler E. J. (2013). The brain reward circuitry in mood disorders. Nat. Rev. Neurosci. 14, 609–625. 10.1038/nrn3381 23942470 PMC3867253

[B179] Schmidt L. M. Hesse M. Lykke J. (2011). The impact of substance use disorders on the course of schizophrenia—a 15-year follow-up study: dual diagnosis over 15 years. Schizophr. Res. 130, 228–233. 10.1016/j.schres.2011.04.011 21592731

[B180] Schmidt M. V. Wang X.-D. Meijer O. C. (2011). Early life stress paradigms in rodents: potential animal models of depression? Psychopharmacology 214, 131–140. 10.1007/s00213-010-2096-0 21086114

[B181] Shand F. L. Degenhardt L. Slade T. Nelson E. C. (2011). Sex differences amongst dependent heroin users: histories, clinical characteristics and predictors of other substance dependence. Addict. Behav. 36, 27–36. 10.1016/j.addbeh.2010.08.008 20833480 PMC2981789

[B182] Shankman S. A. Katz A. C. DeLizza A. A. Sarapas C. Gorka S. M. Campbell M. L. (2014). “The different facets of anhedonia and their associations with different psychopathologies,” in Anhedonia: A Comprehensive Handbook Volume I: Conceptual Issues And Neurobiological Advances, ed. Ritsner M. S. (Dordrecht Springer Netherlands), 3–22.

[B183] Sheridan M. A. McLaughlin K. A. (2014). Dimensions of early experience and neural development: deprivation and threat. Trends Cogn. Sci. 18, 580–585. 10.1016/j.tics.2014.09.001 25305194 PMC4252647

[B184] Shonkoff J. P. Garner A. S. Siegel B. S. Dobbins M. I. Earls M. F. McGuinn L.. (2012). The lifelong effects of early childhood adversity and toxic stress. Pediatrics 129, e232–e246. 10.1542/peds.2011-2663 22201156

[B185] Short A. K. Baram T. Z. (2019). Early-life adversity and neurological disease: age-old questions and novel answers. Nat. Rev. Neurol. 15, 657–669. 10.1038/s41582-019-0246-5 31530940 PMC7261498

[B186] Simpson T. L. Miller W. R. (2002). Concomitance between childhood sexual and physical abuse and substance use problems. A review. Clin. Psychol. Rev. 22, 27–77. 10.1016/s0272-7358(00)00088-x 11793578

[B187] Sinha R. (2008). Chronic stress, drug use, and vulnerability to addiction. Ann. N Y Acad. Sci. 1141, 105–130. 10.1196/annals.1441.030 18991954 PMC2732004

[B188] Smith K. S. Berridge K. C. (2007). Opioid limbic circuit for reward: interaction between hedonic hotspots of nucleus accumbens and ventral Pallidum. J. Neurosci. 27, 1594–1605. 10.1523/JNEUROSCI.4205-06.2007 17301168 PMC6673729

[B189] Smyke A. T. Koga S. F. Johnson D. E. Fox N. A. Marshall P. J. Nelson C. A.. (2007). The caregiving context in institution-reared and family-reared infants and toddlers in Romania. J. Child Psychol. Psychiatry 48, 210–218. 10.1111/j.1469-7610.2006.01694.x 17300560

[B190] Stein M. D. Conti M. T. Kenney S. Anderson B. J. Flori J. N. Risi M. M.. (2017). Adverse childhood experience effects on opioid use initiation, injection drug use, and overdose among persons with opioid use disorder. Drug Alcohol Depend. 179, 325–329. 10.1016/j.drugalcdep.2017.07.007 28841495 PMC5599365

[B191] Strathearn L. Giannotti M. Mills R. Kisely S. Najman J. Abajobir A. (2020). Long-term cognitive, psychological and health outcomes associated with child abuse and neglect. Pediatrics 146:e20200438. 10.1542/peds.2020-0438 32943535 PMC7786831

[B192] Sussman S. Leventhal A. (2014). Substance misuse prevention: addressing anhedonia. New Dir. Youth Dev. 2014, 45–56, 10. 10.1002/yd.20085 24753277 PMC4181563

[B193] Teicher M. H. Samson J. A. Anderson C. M. Ohashi K. (2016). The effects of childhood maltreatment on brain structure, function and connectivity. Nat. Rev. Neurosci. 17, 652–666. 10.1038/nrn.2016.111 27640984

[B194] Tomiyama J. A. Dallman M. F. Epel E. S. (2011). Comfort food is comforting to those most stressed: evidence of the chronic stress response network in high stress women. Psychoneuroendocrinology 36, 1513–1519. 10.1016/j.psyneuen.2011.04.005 21906885 PMC3425607

[B196] Treadway M. T. Buckholtz J. W. Schwartzman A. N. Lambert W. E. Zald D. H. (2009). Worth the ‘EEfRT’? The effort expenditure for rewards task as an objective measure of motivation and anhedonia. PLoS One 4:e6598. 10.1371/journal.pone.0006598 19672310 PMC2720457

[B195] Treadway M. T. Zald D. H. (2013). Parsing anhedonia: translational models of reward-processing deficits in psychopathology. Curr. Dir. Psychol. Sci. 22, 244–249. 10.1177/0963721412474460 24748727 PMC3989147

[B197] van Os J. Kenis G. Rutten B. P. (2010). The environment and schizophrenia. Nature 468, 203–212. 10.1038/nature09563 21068828

[B198] Vazquez V. Giros B. Daugé V. (2006). Maternal deprivation specifically enhances vulnerability to opiate dependence. Behav. Pharmacol. 17, 715–724. 10.1097/FBP.0b013e3280116e6f 17110797

[B199] Vazquez V. Penit-Soria J. Durand C. Besson M. Giros B. Daugé V. (2005). Maternal deprivation increases vulnerability to morphine dependence and disturbs the enkephalinergic system in adulthood. J. Neurosci. 25, 4453–4462. 10.1523/JNEUROSCI.4807-04.2005 15872092 PMC6725024

[B200] Vey L. T. Rosa H. Z. Barcelos R. C. S. Segat H. J. Metz V. G. Dias V. T.. (2016). Stress during the gestational period modifies pups’ emotionality parameters and favors preference for morphine in adolescent rats. Behav. Brain Res. 296, 408–417. 10.1016/j.bbr.2015.08.012 26300452

[B201] Vigorito M. Sclafani A. (1988). Ontogeny of polycose and sucrose appetite in neonatal rats. Dev. Psychobiol. 21, 457–465. 10.1002/dev.420210505 3402668

[B202] Volkow N. D. McLellan T. A. Cotto J. H. Karithanom M. Weiss S. R. B. (2011). Characteristics of opioid prescriptions in 2009. JAMA 305, 1299–1301. 10.1001/jama.2011.401 21467282 PMC3187622

[B203] Wakeford A. G. P. Morin E. L. Bramlett S. N. Howell L. L. Sanchez M. M. (2018). A review of nonhuman primate models of early life stress and adolescent drug abuse. Neurobiol. Stress 9, 188–198. 10.1016/j.ynstr.2018.09.005 30450384 PMC6236515

[B204] Walker C.-D. Bath K. G. Joels M. Korosi A. Larauche M. Lucassen P. J.. (2017). Chronic early life stress induced by limited bedding and nesting (LBN) material in rodents: critical considerations of methodology, outcomes and translational potential. Stress 20, 421–448. 10.1080/10253890.2017.1343296 28617197 PMC5705407

[B205] Whitfield C. L. Dube S. R. Felitti V. J. Anda R. F. (2005). Adverse childhood experiences and hallucinations. Child Abuse Negl. 29, 797–810. 10.1016/j.chiabu.2005.01.004 16051353

[B206] Whitton A. E. Treadway M. T. Pizzagalli D. A. (2015). Reward processing dysfunction in major depression, bipolar disorder and schizophrenia. Curr. Opin. Psychiatry 28:7. 10.1097/YCO.0000000000000122 25415499 PMC4277233

[B207] Widom C. S. Marmorstein N. R. White H. R. (2006). Childhood victimization and illicit drug use in middle adulthood. Psychol. Addict. Behav. 20, 394–403. 10.1037/0893-164X.20.4.394 17176174

[B208] Willner P. Muscat R. Papp M. (1992). Chronic mild stress-induced anhedonia: a realistic animal model of depression. Neurosci. Biobehav. Rev. 16, 525–534. 10.1016/s0149-7634(05)80194-0 1480349

[B209] Wright E. R. Kooreman H. E. Greene M. S. Chambers R. A. Banerjee A. Wilson J. (2014). The iatrogenic epidemic of prescription drug abuse: county-level determinants of opioid availability and abuse. Drug Alcohol Depend. 138, 209–215. 10.1016/j.drugalcdep.2014.03.002 24679840

[B210] Yan C.-G. Rincón-Cortés M. Raineki C. Sarro E. Colcombe S. Guilfoyle D. N.. (2017). Aberrant development of intrinsic brain activity in a rat model of caregiver maltreatment of offspring. Transl. Psychiatry 7:e1005. 10.1038/tp.2016.276 28094810 PMC5545736

[B211] Zald D. H. Treadway M. T. (2017). Reward processing, neuroeconomics, and psychopathology. Annu. Rev. Clin. Psychol. 13, 471–495. 10.1146/annurev-clinpsy-032816-044957 28301764 PMC5958615

[B212] Zhang L. I. Bao S. Merzenich M. M. (2001). Persistent and specific influences of early acoustic environments on primary auditory cortex. Nat. Neurosci. 4, 1123–1130. 10.1038/nn745 11687817

